# Case report: Bilateral spinal neurofibromatosis

**DOI:** 10.3389/fneur.2022.976929

**Published:** 2022-08-12

**Authors:** Ali Baradaran Bagheri, Sepehr Aghajanian, Aliasghar Taghi Doulabi, Mehdi Chavoshi-Nejad, Somayeh Sorouredin Abadi

**Affiliations:** ^1^Department of Neurosurgery, Shahid Madani Hospital, Alborz University of Medical Sciences, Karaj, Iran; ^2^Student Research Committee, School of Medicine, Alborz University of Medical Sciences, Karaj, Iran; ^3^Department of Internal Medicine, Shahid Madani Hospital, Alborz University of Medical Sciences, Karaj, Iran

**Keywords:** spinal neurofibromatosis, nerve sheath tumor, Von Recklinghausen's disease, spinal tumor, neurosurgical oncology, case report

## Abstract

Spinal neurofibromatosis (SNF) is a rare form of Neurofibromatosis in which neurofibromas exist bilaterally throughout all spinal roots. Despite previous attempts made to characterize and classify the disease as a separate clinical form of the disease, the low incidence rate of the disease and scarcity of previous reports calls for further studies and reports to elaborate this clinical entity. The patient in this report was a 36-year-old man presenting with lower limb weakness, unsteady gait, and paresthesia. The patient also presented with multiple cutaneous café-au-lait spots, cutaneous neurofibromas, and a large neurocutaneous neurofibroma of right facial nerve. Magnetic resonance imaging (MRI) of spine revealed bilateral spinal neurofibromas across all spinal cord roots. MRI study of head revealed no abnormalities in the brain and optic tract. The patient fulfilled both NIH criteria as well as revised criteria for NF1. Despite total spinal cord involvement, surgical intervention was withheld from the patient due to high propensity of recurrence as seen with previous attempts in removing peripheral neurofibromas, slow progression of symptoms, and lack of significant pain and impairment. SNF is often described as a form of disease with infrequent presentation of classical NF1 symptoms other than spinal tumors. The case presented here however, presented with several cutaneous neurofibromas and café-au-lait spots. Considering the positive outcome of surgical intervention in a few other reports, the decision to surgically intervene should be left to the clinical judgement of the participating surgeon, patient preference and socioeconomic background in a case-by-case manner.

## Introduction

The Neurofibromatoses are a group of genetic neurocutaneous disorders with an autosomal dominant inheritance pattern and significant morbidity and mortality. These conditions are characterized by dysregulated cell growth in tissues that lead to tumor growth in nerves throughout the body in any age (1, 2). Despite the significant heterogeneity in clinical presentation of the affected individuals and several reports of variants and alternate forms of the disease (2, 3), the neurofibromatoses have been generally classified into three clinical entities, Neurofibromatosis type 1 (NF1, 96% of all cases) and NF2, which are well-characterized based on genetic defects in the relevant genes, tumor type and location, and clinical determinants of each type, and a rare third type (<1% of all NF cases) called Schwannomatosis with distinct mutations in SMARCB1 or LZTR1 genes, but a clinical presentation comparable to NF2 excluding bilateral vestibular schwannomas and an older age of onset (4–8).

NF1, historically known as Von Recklinghausen's disease, affects all races and ethnicities with a reported incidence of 1 in 3,000–1 in 6,000 and an estimated birth incidence of 1/2,558–1/3,333, is caused by mutations in *NF1* gene localized to chromosome 17 (6, 9). Although autosomal dominant pattern of inheritance in both NF types suggests vertical transmission as the primary source of gene mutation, about half of the NF1 cases are represented by *de novo* mutations in the *NF1* sequence (10). The considerable proportion of cases without family history of NF1 reflects the high rate of mutation of *NF1* locus, with the majority of the deletions and mutations being of maternal and paternal origin, respectively (11). The gene product of *NF1*, neurofibromin, is a GTPase-activating protein that acts as a negative regulator of RAS/MAPK pathway (12). Mutations in *NF1* gene result in diminished tumor suppressive properties, RAS hyperactivation, and subsequent upregulation of mTOR and ERK pathways (6), which have also been linked to increased predisposition toward certain tumors and/or malignancies including pheochromocytoma, optic pathway glioma, astrocytomas and malignant gliomas, breast cancer, gastrointestinal stromal tumors, rhabdomyosarcomas, and peripheral nerve sheath neoplasms (13, 14). As such, genetic counseling should be offered to families with NF1 and tailored imaging guidelines have been developed for surveillance based on clinical symptoms (14, 15).

The disease phenotype is characterized by multiple skin pigmentations (café-au-lait macules and lentiginous macules), melanocytic hamartomas of the iris (Lisch nodules), cutaneous (dermal), subcutaneous (peripheral nodular), internal, and/or plexiform neurofibromas, optic gliomas, intellectual disability, skeletal dysplasia, and short stature. Dermal and peripheral nerve neurofibromas comprise the majority of benign tumors in classical NF1 (15–17). While the occurrence of spinal neurofibromas is more restricted in classical disease (36% of the patients, 5% with spinal cord complication), these tumors are more likely to be seen in rare clinical variants of NF1 involving multiple spinal roots (multiple neurofibromas in spinal roots, MNFSR) and bilateral involvement of all spinal nerves (Spinal neurofibromatosis, SNF) (18).

Studies describing SNF have been relatively few and far between, with limited characterization of other manifestations of SNF, uncertainty of the disease prognosis compared to classical NF1, and incomprehensive evaluation of efficacy of therapeutic approaches in tailored management of the disease that is largely related to the low incidence rate, the atypical symptoms, and the asymptomatic nature of the spinal nerve lesions until the later stages of SNF. As such, additional reports describing this distinct phenotype would allow a thorough understanding of this rare clinical entity. In this study, we describe a patient with SNF presenting with bilateral involvement of all spinal roots, neurocutaneous symptoms, and lower limb weakness. The diagnosis, prognosis, and symptomatic treatment of this peculiar form of NF have also been discussed throughout the text to provide further insight regarding this condition.

## Case description

**Figure 1:**
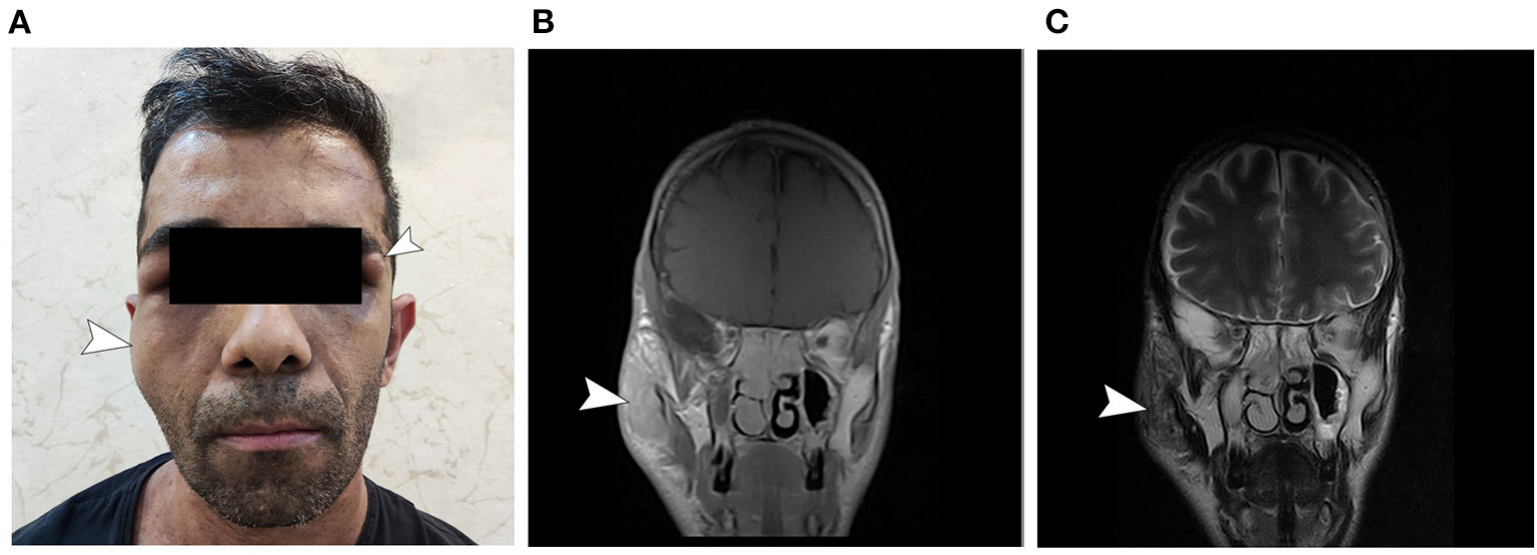
General appearance of the patient. **(A)** Note the presumed neurofibroma of the right facial nerve and the neurofibroma in the left periorbital region. The patient had multiple café-au-lait spots on his abdomen and thorax (not shown). **(B)** T1 gadolinium-enhanced and **(C)** T2-weighted MRI sections corresponding to the right-side presumed facial neurofibroma.

The patient described is a 37-year-old man presenting with lower limb weakness, unsteady gait, and paresthesia. Upon inspection, the patient also presented with multiple large cutaneous café-au-lait spots, cutaneous neurofibromas, and a large neurofibroma of right facial nerve, which according to the patient, recurred over the years after its primary resection when the patient was 7 years old (Figure 1). He reported a history of cutaneous neurofibromas in his maternal grandfather and café-au-lait macules in patient's mother and uncle, indicating maternal origin of NF gene mutation. According to the patient, he did not experience learning difficulties throughout his childhood. The patient had mild weakness of the lower limbs since prepubescence that did not significantly affect his physical activity until a year before visiting the current physician, when the symptoms worsened leading to imbalance, unsteady gait, and inability to continue intense manual labor in his previous occupation.

Physical examination revealed normal range of motion in the spine, shoulder, hip, and knee joints (negative McMurray circumduction and drawer tests), normal bowel and bladder function, unremarkable cranial nerves (no focal signs) and ophthalmological exam (no Lisch nodules in iris), and negative Babinski sign; but brisk (+3) deep tendon reflexes and positive left leg straight leg raising test. Audiogram and tympanometry results revealed no hearing abnormalities. The patient fulfilled both NIH criteria and revised criteria for NF1 (8, 13). Genetic testing was unavailable at the time of patient evaluation and was omitted due to considerable financial burden and compatible clinical presentation. Written informed consent to publish clinical data and findings was obtained from the patient.

**Figure 2:**
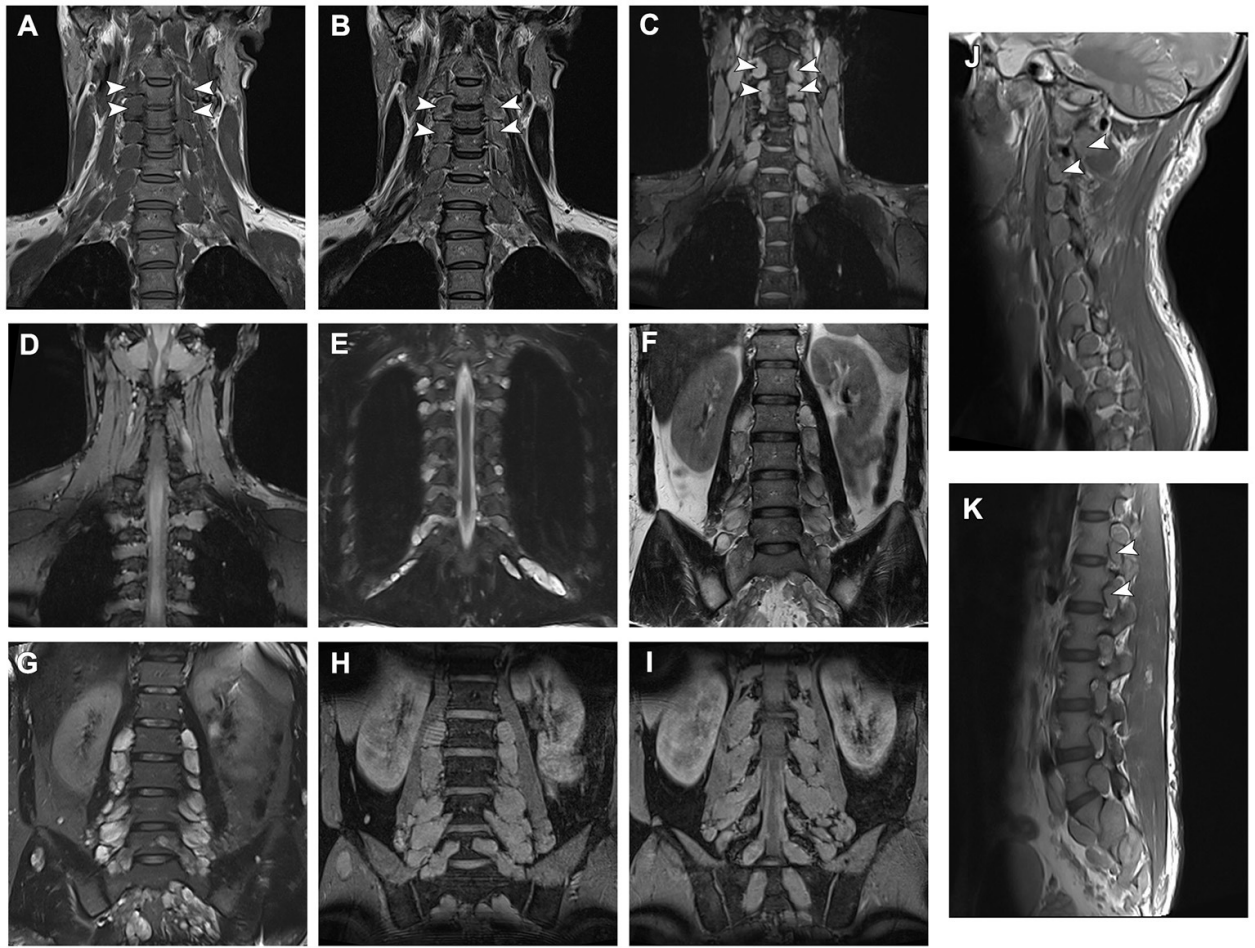
Magnetic resonance imaging data of the patient and neurofibromas across all spinal roots. **(A,B)** T1- and T2-weighted and **(C)** Multiple Echo Data Image Combination (MEDIC) coronal sections of the cervical spine MRI demonstrating presumed neurofibromas affecting all nerve roots. **(D,E)** MEDIC and T2 sequence images of thoracic neurofibromas. **(F)** Fast spin echo and **(G)** turbo inversion recovery magnitude T2-weighted and **(H,I)** MEDIC coronal sections of the lumbar vertebrae demonstrating lumbar and sacral neurofibromas. **(J,K)** Sagittal T1 sequences of the spine demonstrating the proximal extension of the tumors.

Magnetic resonance imaging (MRI) of spine revealed the presence of presumed neurofibromas bilaterally through all spinal cord roots (Figure 2). Brain MRI revealed no abnormalities in cortical or underlying structures.

## Discussion

SNF could be described as a distinct clinical entity in which bilateral neurofibromas in all spinal nerves and/or spinal roots are the main clinical presentation of the patients with a less frequent pattern of other NF1 manifestations such as Lisch nodules, changes in muscle tone, or skeletal dysplasia. As a corollary, only a minority of SNF cases could completely satisfy the NF1 diagnostic criteria (18). Dermal neurofibromas are less common in SNF than in NF1 despite extensive peripheral nerve enlargement extending from each spinal nerve. As most reports described this phenotype in segregated families, SNF was initially referred to as hereditary/familial spinal neurofibromatosis. However, missense mutations in *NF1* have been observed to be significantly higher in SNF. As such, individuals harboring *de novo NF1* missense mutations may develop SNF in a family without a history of the disease. Nevertheless, obtaining baseline MRI of the entire CNS to screen for asymptomatic tumors in newly diagnosed and asymptomatic individuals with NF1 is not currently recommended (14). Accordingly, imaging studies should be reserved for individuals demonstrating abnormal neurological examination, progressive symptoms of cord compression and polyneuropathy, or unexplained neurological deficits using localized imaging with multiple MR sequences (14).

At present, the treatment strategies revolve around symptomatic relief of disease manifestations and improving quality of life. Despite extensive research, recent clinical trials have demonstrated that pharmacological interventions have diminished ability to reduce tumor size in plexiform neurofibromas. Furthermore, spontaneous regression of neurofibromas is rarely seen in clinical settings (14).

The mainstay therapeutic approach for symptomatic neurofibromas are surgical excision of certain tumors which cause significant morbidity. Spinal cord compression symptoms and spinal deformity have been valid indications for anterior and/or posterior decompression with or without fusion/arthrodesis, and complete or partial resection of neurofibromas in classical NF1 (19, 20). However, bilateral involvement of all vertebrae in SNF restricts less invasive surgical approaches such as hemilaminectomy or tumor resection without instrumentation. Multilevel bilateral laminectomies are also prone to significant destabilization of spinal column, which may result in several postoperative complications (20). The authors therefore believe that surgical intervention should be reserved for cases of severe disability and to be limited to symptomatic lesions. A previous report found that the majority of preoperative symptoms improved in patients with non-NF2 spinal neuromas compared to their NF2 counterparts, with a low 5-year recurrence rate of 10.7%. However, the scarcity of NF1 cases in the study precludes definitive conclusions on the prognosis and recurrence of neurofibromas in SNF (21).

While classical NF1 symptoms is less frequently seen in SNF, this case presented with several cutaneous neurofibromas, café-au-lait spots, and movement disorder. It is of note to say that symptomatic SNF reportedly consists only 1.6% of all NF1 cases (9, 18). Although there was total spinal cord involvement with intradural extension of the tumors in several spinal levels in this case, surgical intervention was withheld from the patient due to high propensity of recurrence as seen with previous attempts in removing peripheral neurofibromas, slow progression of symptoms, financial burden on the patient in the context of economic inequality caused by NF1 (22), lack of significant pain or impairment in daily activities, and risk of complications. Considering the positive outcome of surgical intervention in a few other reports in improving patient quality of life and symptomatic relief without evidence of short term recurrence, the decision to surgically intervene should be left to the clinical judgement of the participating surgeon, patient preference and background in a case-by-case manner. At the time of writing this work, the conservative approach to the spinal tumors in this case was approved and well-tolerated by the patient.

## Data availability statement

The raw data supporting the conclusions of this article will be made available by the authors, without undue reservation.

## Ethics statement

Ethical review and approval was not required for the study on human participants in accordance with the local legislation and institutional requirements. The patient provided their written informed consent to participate in this study. Written informed consent was obtained from the patient for the publication of any potentially identifiable images or data included in this article.

## Author contributions

AB designed and supervised the study and was the primary physician of the patient. SA and AT acquired data from the patient, investigated the patient history, and clinical data. SA and AB analyzed the data. SA and MC-N contributed with visualization and drafting the manuscript. SA and SSA edited the manuscript for clarity and scientific accuracy. All authors contributed to the article and approved the submitted version.

## Conflict of interest

The authors declare that the research was conducted in the absence of any commercial or financial relationships that could be construed as a potential conflict of interest.

## Publisher's note

All claims expressed in this article are solely those of the authors and do not necessarily represent those of their affiliated organizations, or those of the publisher, the editors and the reviewers. Any product that may be evaluated in this article, or claim that may be made by its manufacturer, is not guaranteed or endorsed by the publisher.
